# MULTIPLE NEUROENDOCRINE NEOPLASIA IN A PATIENT WITH TYPE I NEUROFIBROMATOSIS (NF1): REPORT OF A NEW MUTATION (*NF1*, EXONS 2-30 DELETION) AND LITERATURE REVIEW

**DOI:** 10.1590/0102-672020220002e1702

**Published:** 2023-01-09

**Authors:** Danilo Toshio KANNO, Roberta Lais Mendonça de MATTOS, Fábio Guilherme CAMPOS, Rayama Moreira SIQUEIRA, Rita Barbosa de CARVALHO, Carlos Augusto REAL MARTINEZ

**Affiliations:** 1 Universidade São Francisco, Graduate Program in Health Sciences – Bragança Paulista (SP), Brazil;; 2 Universidade Estadual de Campinas, Department of Surgery, Gastrocenter – Campinas (SP), Brazil.

**Keywords:** Neurofibromatosis 1, Genes, Colon, Neurilemmoma, Neuroendocrine Tumors, Neurofibromatose 1, Genes, Colo, Neurilemoma, Tumores Neuroendócrinos

## Abstract

**BACKGROUND:**

Plexiform neurofibromas represent a common neoplasia of type 1 neurofibromatosis in which neurofibromas arise from multiple nerves involving connective tissue and skin and rarely affect the colon and rectum. Co-occurrence of plexiform neurofibromas, neuroendocrine tumors with primary involvement of the rectum, and medullary thyroid carcinoma in patients with neurofibromatosis type 1 is a previously undescribed condition. The aim of this manuscript was to present a case of primary plexiform neurofibroma and neuroendocrine tumors of the upper rectum in a patient with neurofibromatosis type 1 whose genetic sequencing found a novel mutation in the neurofibromatosis type 1 gene and to review the literature.

**CASE REPORT:**

A 49-year-old woman with a familial history of neurofibromatosis type 1 complained of abdominal cramps for 6 months. She had previously been submitted for a total thyroidectomy due to medullary thyroid carcinoma. She was submitted to a colonoscopy, which identified a submucosa lesion located in the upper rectum. The patient was referred for a laparoscopic rectosigmoidectomy, and the histopathological study of the surgical specimen identified two different tumors. An immunohistochemical panel was done for histopathological confirmation of the etiology of both lesions. The results of the panel showed intense immunoexpression of S100 protein in the largest and superficial lesion, as well as positivity for chromogranin and synaptophysin in the minor and deep lesion confirming the diagnosis of rectal plexiform neurofibromas concomitant with neuroendocrine tumors. The proliferative activity rate using Ki-67 antibodies showed that both tumors had a low rate of mitotic activity (<1%). Genetic sequence panel identified an undescribed mutation in the neurofibromatosis type 1 gene (deletion, exons 2–30). The patient’s postoperative evolution was uneventful, and she remains well, without recurrence, 3 years after surgery.

**CONCLUSION:**

The co-occurrence of medullary thyroid carcinoma, plexiform neurofibromas, and neuroendocrine tumors of the rectum in patients with neurofibromatosis type 1 is an exceptional and undescribed possibility, whose diagnosis can be confirmed by the immunohistochemical staining and genetic panel.

## RESUMO

**RACIONAL:**

Os neurofibromas plexiformes representam uma neoplasia frequente na neurofibromatose tipo 1 onde os neurofibromas surgem de múltiplos nervos envolvendo, também, o tecido conjuntivo e a pele raramente acometendo o cólon e o reto. A co-ocorrência de neurofibromas plexiformes, tumores neuroendócrinos, com envolvimento primário do reto, e carcinoma medular de tireoide em pacientes com neurofibromatose tipo 1 é uma condição ainda não descrita. O objetivo deste manuscrito é descrever um caso de neurofibromas plexiformes e tumores neuroendócrinos primários do reto superior, em paciente com neurofibromatose tipo 1, cujo sequenciamento genético identificou uma nova mutação no gene neurofibromatose tipo 1 e revisar a literatura sobre o tema.

**DESCRIÇÃO DO CASO:**

Mulher de 49 anos, com história familiar de neurofibromatose tipo 1 queixava-se de cólicas abdominais há seis meses. Tinha sido previamente submetida a tireoidectomia total por carcinoma medular de tireoide. Foi submetida a colonoscopia, que identificou lesão submucosa localizada no reto superior. A doente foi encaminhada para retossigmoidectomia laparoscópica, cujo estudo histopatológico do espécime cirúrgico identificou dois tumores distintos. Realizou-se painel imuno-histoquímico para confirmação histopatológica da etiologia de ambas as lesões. O resultado do painel mostrou intensa imunoexpressão da proteína S-100 da lesão superficial e de maior tamanho,e positividade para cromogranina e somatofisina da lesão mais profunda e de menores dimensões confirmando o diagnóstico de neurofibromas plexiformes retal e tumores neuroendócrinos. O índice de atividade proliferativa utilizando o anticorpo ant-Ki-67 mostrou que ambos os tumores apresentavam baixa índice mitótico (<1%). O painel genético identificou mutação no gene neurofibromatose tipo 1 (deleção, exons 2–30) ainda não descrita. A doente apresentou evolução pós-operatória sem intercorrências, permanecendo bem, sem recorrência, três anos após a cirurgia.

**CONCLUSÕES:**

A co-ocorrência de carcinoma medular de tireoide, neurofibromas plexiformes e tumores neuroendócrinos do reto em pacientes com neurofibromatose tipo 1 é uma possibilidade excepcional e ainda não descrita, cujo diagnóstico pode ser confirmado pela coloração imuno-histoquímica e painel genético.

## INTRODUCTION

Neurofibromatosis type 1 (NF1; OMIM #162200), previously known as von Recklinghausen’s disease, is one of the most common autosomal dominant hereditary tumor syndromes, occurring in approximately 1 in 2,500–3,000 births, with a virtual penetrance of 100% by the age of 5 years^30,32^. The syndrome is caused by a loss of function due to a mutation in the neurofibromin 1 (*NF1*) gene (OMIM #613113) that encodes a tumor-suppressed protein named neurofibromin^29^. This cytoplasmic protein controls cellular proliferation and is expressed in many types of tissue, including nerve cells, oligodendrocytes, and Schwann cells that surround nerves^7^. The central nervous system, skin, and bone tumors are the major features of NF1 and affect patients who carry a 60% lifetime risk of developing a malignancy, mainly of the nervous system^10^. Benign tumors like neurofibromas are frequent in NF1 patients, and plexiform neurofibromas (PNs) are pathognomonic of the disease^10^. Visceral involvement in patients with NF1 is uncommon and gastrointestinal manifestations can occur in about 5–25% of cases^3,8,25^. Intra-abdominal (gastrointestinal tract or retroperitoneal) abnormalities in patients with NF1 necessitating surgical treatment are difficult to estimate, and their incidence varies from 2.5 to 25%. Usually, abdominal manifestations in patients with NF1 can be classified into five different categories: benign or malignant neurogenic tumors, neuroendocrine tumors (NETs), gastrointestinal stromal tumors (GISTs), tumors of embryonic origin, and adenocarcinoma at different gastrointestinal sites, vascular lesions, and juvenile-like mucosal gastrointestinal polyps^10,17,23^.

Although neurofibromas are the hallmark of the disease, patients affected by NF1 have an increased risk of developing tumors other than neurofibromas, like schwannomas, Schwann cell hamartoma, PNs, and especially tumors of endocrine origin^12,21,36^. The presence of PNs is pathognomonic of NF1 syndrome, and the presence of synchronous GISTs and NETs in the background of disease is an uncommon possibility^28^. NF1 can also be present in patients with type 2 multiple endocrine neoplasia (MEN2), but this concomitance co-occurs extremely rarely^9^. In this MEN2, medullary thyroid carcinoma (MTC) can be the only manifestation, or it can be associated with pheochromocytoma and hyperparathyroidism in subtype 2A (MEN2A), and with pheochromocytoma, mucosal neuromas, prolonged facies, muscular-skeletal abnormalities, ocular abnormalities, and Marfanoid habitus in subtype 2B (MEN2B)^11^.

Co-occurrence of MTC, PNs, and NET localized in the upper rectum in patients with NF1 syndrome is a condition not yet described. Thus, the aim of this study was to describe the case of a patient with NF1, with a new variant of *NF1* mutation (deletion, exons 2–30), previously submitted to a thyroidectomy due to MTC, in whom we identified a PN associated with a NET of the upper rectum and review the literature.

## CASE REPORT

**Figure 1.**
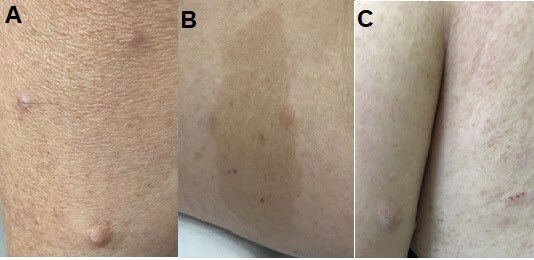
A: Multiple skin neurofibromas (arrows); B: Café-au-lait spot; C: Axillary freckling.

**Figure 2.**
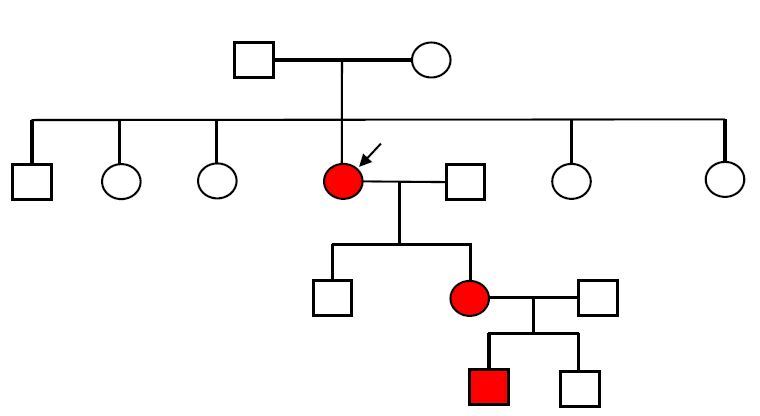
Familial heredogram (arrow shows the patient of this report).

A 49-year-old woman sought specialized care due to abdominal cramps, a change in her bowel habits, tenesmus, and weight loss of 4 kg in the past 6 months. A physical examination showed multiple café-au-lait spots on the trunk and limbs, numerous skin tumors, freckles in the inguinal and axillary regions (Figure 1), and Lisch nodules in both irises. The familiar heredogram confirmed that the patient allowed the clinical diagnosis of NF1, and she has a daughter and a grandson with a characteristic cutaneous manifestation of NF1 (Figure 2). She had undergone a total thyroidectomy 8 years ago, whose histopathological examination confirmed the diagnosis of MTC. She denied a familiar history of thyroid neoplasia. The abdomen and digital rectal examinations were found to be normal, and no palpable mass was identified. She had a positive fecal blood test, and she underwent a colonoscopy to investigate the positivity of the fecal blood test. Colonoscopy revealed an extrinsic compression of the colon walls due to an oval lesion measuring about 6 cm in diameter and occupying 50% of the colon lumen in the upper rectum, 15 cm from the anal margin. Colonoscopy also showed a similar second submucosal tumor measuring 2.0 cm in its largest diameter and located just above the first described lesion. Both lesions were covered by mucosa with edema and mild hyperemia, from which fragments of the major tumor were collected by means of a deep biopsy of the lesion for histopathological study. To assess a possible tumor located outside the colon wall, magnetic resonance imaging (MRI) of the abdomen was performed, which confirmed only the presence of the expansive and infiltrative formation located in the wall of the intraperitoneal rectum, measuring 6.0×2.0 cm in size. The tumor was isointense to muscle on T1-weighted images with a target signal on fluid-sensitive sequences, with variable enhancement without early arterial intensification. The lesion involved the mucous and submucosal layers of the rectal wall, with a loss of definition of the muscle layer itself without signs of extension beyond the serous layer. There was no increase in the regional lymph nodes. The adrenal glands were normal. Carcinoembryonic antigen (CEA) level was 0.9 ng/mL and Ca 19-9 antigen was 6 U/mL.

Histopathological examination of colonoscopy fragments showed colonic mucosa with linear colonic glands and preservation of the goblet cell population. Exaggerated proliferation of elongated or spindly cells, with oval nuclei, blunt edges, and eosinophilic cytoplasm, spreads in multiple directions, forming intersecting cellular bundles in the chorion. These cells had a low mitotic index in high magnification fields (1/10 fields). Cell atypia was not identified.

**Figure 3.**
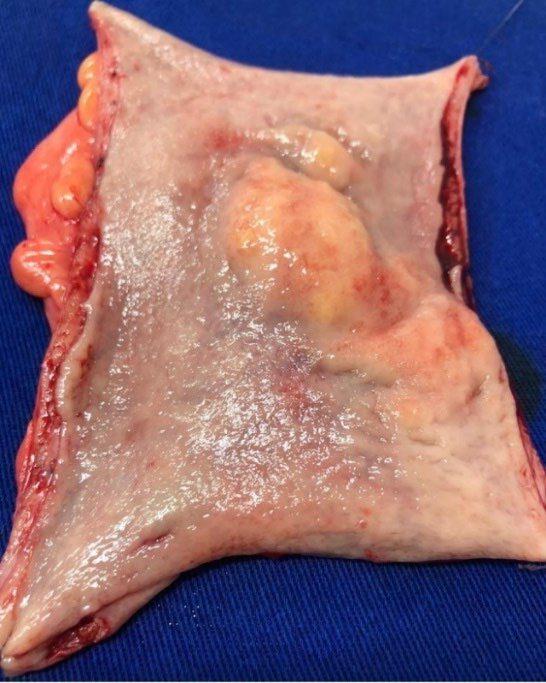
Sigmoid colon specimen with two yellowish submucosal tumors covered by slightly hyperemic mucosa.

**Figure 4.**
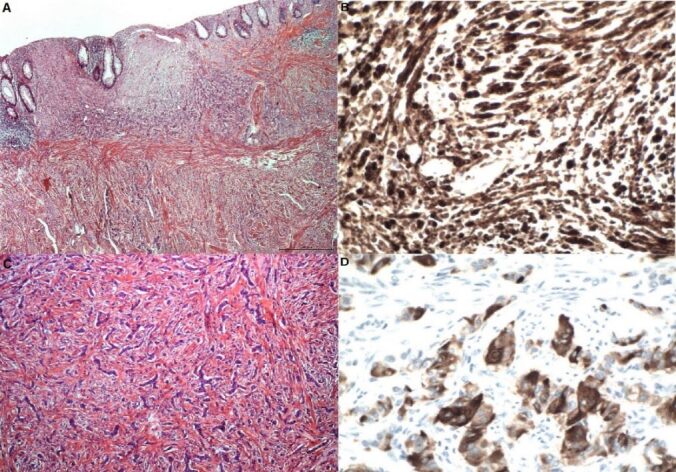
A: Elongated or spindle cells, with oval or fusiform nuclei, blunt edges, and eosinophilic cytoplasm, spread in multiple directions forming intersecting cellular bundles suggestive of PN of (HE 100´); B: Strongest staining for S100 protein (100´); C: Epithelial neoplasm composed of small cells, with round nuclei and slightly granular cytoplasm suggestive of low-grade NET (200´); D: Positive cellular staining for chromogranin A (400´).

With the diagnosis of neurofibroma of the rectum, the patient underwent a laparoscopic anterior resection of the rectum and sigmoid with primary mechanical anastomosis as proposed anteriorly^6^. She presented evolution without complications and was discharged on the third postoperative day. During the procedure, two skin nodules were resected for histopathological study. The anatomopathological study of the resected specimen showed the presence of a yellowish tumor in the rectal wall, with a diameter of 6 cm, covered by normal mucosa with areas of hyperemia and edema, and a second tumor with diameter of 2 cm, located just above the major tumor (Figure 3). Histopathological study of the surgical specimen showed mesenchymal proliferation consisting of elongated cells, with wavy nuclei and loose stroma, affecting the mucosa and submucosa. In the immunohistochemical study, irregularly expanded nerve fascicles were found in the submucosa, Auerbach’s myenteric plexus, and pericolic adipose tissue (Figure 4A). This mesenchymal proliferation was positive for S100 (polyclonal) and SOX10 (BC34) and negative for CD117, desmin, CD34, smooth muscle actin (1A4), GLUT1, and claudin-1 (Figure 4B). Concurrently, an epithelial proliferation consisting of a monotonous population of cells with rounded nuclei and eosinophilic cytoplasm was identified in a trabecular arrangement (Figure 4C). This epithelial proliferation presents a strong immunostaining to chromogranin A (DAK-A3), synaptophysin (DAK-SYNAP), and cytokeratin (AE1/AE3), confirming the presence of NET (Figure 4D). The Ki-67 marker showed that both tumors have a low rate of cell proliferation (1%). Table 1 summarizes the results of the immunohistochemical panel used. These findings were compatible with the PN of the rectum, associated with a well-differentiated NET. The histopathological study of the two removed skin nodules confirmed the diagnosis of cutaneous neurofibroma.

**Table 1.** Immunohistochemical panel of the resected colonic specimen.

Antibody	Clone	Result
Mesenchymal cell spindle neoplasia (schwannoma)		
Desmin	D33	Negative
CD34	QBEnd10	Negative
Actin	1A4	Negative
CD117	YR145	Negative
GLUT-1	SPM498	Negative
Claudin-1	Polyclonal	Negative
S100 protein	Polyclonal	Positive
SOX10	BC34	Positive
Ki-67	MIB1	<1%
Epithelial atypical proliferation (NET)		
Chromogranin A	DAK-A3	Positive
Synaptophysin	DAK-SYNAP	Positive
Cytokeratin 40, 48, 50, and 56 kDa	AE1/AE3	Positive
Ki-67 (NET)	MIB1	1%

Due to the diagnosis of NF1, to clarify the type of mutations on the *NF1* gene, the patient was submitted to sequence analysis and deletion/duplication testing of the 84 genes (Invitae Multi-Cancer Panel, San Francisco, CA, USA), which included *NF1* (MedGen UID: 18013). One pathogenic variant was identified in *NF1* (deletion, exons 2–30) heterozygous, classified as pathogenic. This variant is an in-frame deletion of the genomic region encompassing exons 2–30 of the *NF1* gene. It preserves the integrity of the reading frame. The region of the *NF1* gene that includes exon(s) 2 and 9–12 has been determined to be clinically significant^41^. It is important to note that this variant has not been reported in the literature in individuals with NF1-related conditions.

The genetic panel did not find mutations in the *RET* gene, moving away from the possibility of MEN2, but identified a variant of uncertain significance (VUS) in the *POLE* gene (*POLE*, exon 14, c.1370C>T, p.Thr457Met), heterozygous. This sequence change replaces threonine with methionine at codon 457 of the POLE protein. The threonine residue is moderately conserved, and there is a moderate physicochemical difference between threonine and methionine. This variant is not present in population databases (ExAC no frequency). This variant has not been reported in the literature in the germline of individuals with POLE-related conditions. ClinVar contains an entry for this variant (variation ID: 240390). Algorithms developed to predict the effects of missense changes on protein structure and function are either unavailable or do not agree on the potential impact of this missense change. In summary, the available evidence is currently insufficient to determine the role of this variant in disease. Therefore, it has been classified as a VUS.

Currently, the patient is feeling well 3 years after surgery and is having an annual MRI of the skull, chest, and abdomen to screen for the appearance of new PNs or NET in other sites.

## LITERATURE REVIEW

NF1 is one of the most common inherited cancer syndromes^32^. Recent evidence revealed that NF1 is a much more common disease than previously thought, with a birth incidence of 1:2000 and a prevalence of 1:4000^39^. It is an autosomal dominant disorder; however, sporadic inheritance has been described. The syndrome is caused by a loss of function due to a mutation in the *NF1* gene^29^. The *NF1* gene is located on chromosome 17q11.2 and encodes a cytoplasmic protein named neurofibromin 1, which controls cellular proliferation through the p21, RAS, and MAP kinase pathways^1,10^. The *NF1* has a high mutation rate in humans, and only around 50% of NF1 mutations are familial, with the remainder occurring *de novo* without a previous familiar history of the disease^10^. The encoded protein has a GTPase-activating domain that interacts with RAS and converts active RAS-GTP to its inactive form^21^. Biallelic inactivation of the *NF1* gene results in complete loss of functional neurofibromin 1 activity. Penetrance is complete, however, expression of *NF1* is highly variable, depending on the type of mutation (nonsense, frameshift or splice mutations, or deletions are the most common), the time at which the mutation occurs, and the presence of molecular alterations in the associated genes^1,10^.

The diagnosis of NF1 is essentially clinical and based on the guidelines proposed by the National Institute of Health Consensus established in 1988 (Table 2)^24^. Patients with NF1 present skin neurofibromas, PNs, café-au-lait macules, axillary or inguinal freckling, optic gliomas, Lisch nodules (pigmented hamartomas nodular aggregates of dendritic melanocytes affecting the iris), and bony dysplasia^31,43^. Two of the following diagnostic criteria should be fulfilled to make a diagnosis of NF1^10^.

**Table 2.** Diagnostic criteria for type 1 neurofibromatosis (von Recklinghausen’s disease).

Two or more criteria are required for diagnosis
• Six or more café-au-lait macules (>0.5 cm in children or >1.5 cm in adults)
• Two or more cutaneous/subcutaneous neurofibromas or PNs
• Axillary or groin freckling
• Optic pathway glioma
• Two or more Lisch nodules (iris hamartoma seen on slip lamp examination)
• Bony dysplasia (sphenoid wing dysplasia, bowing of long bone ± pseudarthrosis)
• One first degree relative with NF1

NIH consensus development conference 1988^24^.

NF1 patients are at an increased risk of malignancy and vascular malformations and have a life expectancy of about 10–15 years shorter than the general population^5^. The most common abdominal manifestations of patients with NF1 comprehend five categories^3^. The first category is represented by benign or malignant neurogenic tumors, most of them are neurofibroma, but also by PNs or malignant nerve sheath tumors. NET represents the second category and can be of the carcinoid type, mainly localized in the periampullary region of the duodenum, somatostatinoma, pheochromocytoma, paraganglioma, and GIST^17^. The third category includes GIST, a mesenchymal neoplasm, arising from cells of Cajal of the myenteric plexus, and these are probably underdiagnosed with possible confusion with other tumors such as neurofibroma^17^. Embryonal tumors, such as neuroblastoma, rhabdomyosarcoma, or Wilms’ tumors, represent the fourth group. Finally, adenocarcinoma at different gastrointestinal sites, vascular lesions (renal artery stenosis, aneurysms, and arteriovenous fistulas), and juvenile-like mucosal gastrointestinal polyps represent the last category (Table 3)^1,24,43^.

**Table 3.** Classification of abdominal neoplasm associated with NF1.

Neurogenic neoplasms
Solitary neurofibromas
Plexiform neurofibroma
Diffuse mucosal/submucosal neurofibromatosis
Ganglioneuromatosis
Malignant peripheral nerve sheath tumor
**Interstitial cells of Cajal lesions**
Gastrointestinal stromal tumors (GISTs)
Multifocal
Solitary
Minute incidental GISTs tumor
Interstitial cells of Cajal hyperplasia
**Neuroendocrine tumors**
Pheochromocytoma
Neuroendocrine neoplasms
Somatostinoma
**Embryonal**
Rhabdomyosarcoma
**Miscellaneous**
Adenocarcinoma at different gastrointestinal sites
Vasculopathy
Juvenile-like mucosal gastrointestinal polyps

Based on Dare et al.^10^; GIST: gastrointestinal stromal tumor; NF1: type 1 neurofibromatosis.

It is estimated that between 5 and 25% of patients with NF1 will develop intra-abdominal (gastrointestinal or retroperitoneal) neoplastic manifestations^10^. Intra-abdominal tumors may be benign or malignant. However, even benign neoplasm can determine major complications related to the invasion of organs and principal structures of the abdomen^10^. The relative risk for the development of tumors in the digestive tract in patients with NF1 is 8.2 for bile duct tumors, 3.8 for liver, 3.4 for pancreas, 3.3 for esophagus, 2.8 for stomach, and 2.0 for colon. When considering only the lower digestive tract, this risk is 2.4, 2.0, and 1.2 for tumors located at the rectosigmoid junction, colon, and rectum, respectively^35^.

Neurofibromas are the most common type of peripheral nerve sheath tumors in patients with NF1^10^. Neurofibroma is a tumor of the peripheral nervous system and occurs most commonly in the extremities^2^. They are benign nerve sheath tumors of the peripheral nervous system, consisting of a mixture of cell types including Schwann cells, perineural-like cells, mast cells, and fibroblasts^10^. Neurofibromas may appear as focal growths or extend along nerves, involving multiple fascicles, where they are defined as PN, as occurs in the patient of this report. The presence of PN is highly specific to NF1 and they are originating from the neural plexus and are often multiple^14^. PNs occasionally have the potential for malignant transformation into malignant peripheral nerve sheath tumors (MPNST), a soft-tissue sarcoma that is typically high grade with a propensity for distant metastasis^42^. PNs occur in up to 30% of cases of NF1-affected patients, most frequently in the craniomaxillofacial region and rarely compromised the gastrointestinal tract^1,37^. In the abdomen, PNs are reported to affect predominantly the small bowel, retroperitoneum, and, less frequently, the colon^19^. Malignant progression of the PNs is generally considered the main cause of mortality and can develop in 2–16% of patients. Thus, when the diagnosis of gastrointestinal PNs is done, surgical resection should be considered to avoid the possibility of malignant transformation. Symptoms can be nonspecific and relate to tumor growth (pain, especially along the palpable abdominal mass, or bleeding if there is mucosal involvement)^10^. The patient of this report has a previous diagnosis of NF1 and presents abdominal cramps, change of her bowel habits, tenesmus, weight loss, and positive fecal blood test signals and symptoms common in colorectal neoplasia. During the colonoscopy, the finding of a submucosal mass covered by mucosa practically excludes the diagnosis of rectal adenocarcinoma. The biopsy performed at the depth of the lesion during colonoscopy allowed the histological diagnosis of the PNs, however, not identifying the NET.

MRI and positron emission tomography (PET/CT) are imaging tests used to identify PN at risk of malignant transformation to MPNST. PNs are isointense to muscle on T1-weighted images, and demonstrate a target sign on fluid-sensitive sequences, with variable enhancement without early vascular enhancement, as occurred in the patient of this report ^10^. Tumor depth in the colon wall, the presence of necrosis in more than 25% of the lesion, and tumors larger than 5 cm in their longest axis are more common in MPNST^34^. The diffuse and heavy sequences show significantly less diffusivity in MPNST when compared to benign PNs^40^. The presence of edema around the lesion is a more common finding in malignant lesions. PET/CT shows great sensitivity and good specificity in the differential diagnosis between benign and malignant lesions^38^. Generally, benign lesions have lower mean standard uptake values (SUV) when compared to malignant lesions. SUV values greater than 3.5 are generally highly suggestive of malignant transformation^10^. Unfortunately, we were unable to perform PET/CT on the patient in the present report.

The definitive diagnosis of PNs is better made by means of immunohistopathologic examination of the operative specimen. In the patient of this report, the deep biopsy performed through the rectal mucosa during colonoscopy made it possible to suspect rectal PNs by conventional histological and immunohistochemical studies. However, the histological and immunohistochemical studies of the biopsy did not identify the NET. Then, deep biopsy of several sites of the lesion is necessary for diagnosis of the lesions as well as to evaluate malignant transformation. Mostly, only the examination of the resected surgical specimen can identify the malignant transformation or, as occurred in the patient in this report, identify the coexistence of a second neoplasm. The immunohistochemical assay is essential to confirm the histological origin of these tumors. The immunohistochemical study of the patient was positive for S100 protein, SOX10, and negative for desmin, actin CD34, CD117, GLUT-1, and claudin-1, excluding the possibility of smooth muscle tumors, GIST, vascular abnormalities, and adenocarcinomas. The positivity to S100 protein is an important marker of Schwann cells and is useful for evaluating nerve sheath tumors and is employed to differentiate between schwannoma (stains all cells) and PNs (mixture of positive and negative cells). SOX10 is a transcription factor known to be crucial in the development and survival of Schwann and related cells and particularly useful to identify neurofibromas of the gastrointestinal tract^27^. Colorectal PNs are reported as benign in most cases, but the possibility of malignant transformation has been described. They are characterized by a low rate of mitosis, the absence of atypical mitotic figures, and nuclear hyperpigmentation^10^. The degree of aggressiveness depends on the Ki-67 index and the mitotic index, and it is recommended as an indicator of malignancy. A value of less than 3% correlates with lesser tumor aggressiveness, and a value of more than 10% is considered malignant^31^. Then, in the patient of this report, the immunohistochemical study of the rectal-resected specimen confirmed the diagnosis of PNs due to the low rate of cell proliferation (Ki-67, 1%).

Gastrointestinal NET is commonly associated with MEN1 syndrome. NET is most common in NF1 patients than in the general population and shows a predilection for small bowel, particularly in peri-ampullary duodenum^15^. NET in patients with NF1 has a similar rate of malignant degeneration as in the general population^10^. This tumor is frequently of the grade 1 (well-differentiated) neoplasia in patients with NF1 syndrome, as seen in the patient of this report. Somatostatinomas were the most common tumor type of NET in patients with NF1 syndrome, responsible for 40% of cases. NF1-associated somatostatinomas are characteristically duodenal, are rarely associated with somatostatinoma syndrome, and are less likely to metastasize as compared with sporadic somatostatinomas^15^. Colonic compromise by somatostinomas is uncommon in NF1 patients, particularly when PNs are present. In this report, an immunohistochemical study of colorectal tumors usually shows a strong tissue expression of synaptophysin and chromogranin A^15^. The cytokeratin staining shows the epithelial origin of the NET, and the presence of positive Ki-67 in less than 3% of the cells suggest a low-grade (grade 1) NET.

To identify the site of the mutation of the *NF1* gene, to evaluate the status of the *RET* gene due to previous diagnosis of an MTC, and to rule out the possibility that the patient in this report could be an indecisive case of a syndrome associated with MEN2, we performed a genetic panel. This test evaluated 84 genes for variants (genetic changes) that are associated with genetic disorders. Genetic testing, when combined with family history and other medical results, may provide information to clarify individual risk, support a clinical diagnosis, and assist with the development of a personalized treatment and management strategy. Mutations of the *NF1* gene are associated with autosomal-dominant NF1 (MedGen UID: 18013), neurofibromatosis-Noonan syndrome (NFNS) (MedGen UID: 419089), and Watson syndrome (MedGen UID: 107817). There is an increased risk for central nervous system neoplasms^13,18,22^ and other malignancies, including breast, GIST, and soft-tissue sarcomas^16,20^. Additionally, evidence of varying degrees suggests a possible association between the *NF1* gene and several types of epithelial and stromal tumors^3,11,36^. NF1 is highly variable in its expression, even among affected members of the same family. Biological relatives have a chance of being at risk for NF1-related conditions and should consider testing if clinically appropriate. The genetic panel realized in the patient of this report identified a mutation variant that has not been reported in the literature in individuals with NF1-related conditions (deletion, exons 2–30). These regions of the *NF1* gene that includes exon(s) 2 and 9–12 have been determined to be clinically significant. Therefore, deletions that encompass these regions are likely to disrupt protein function and cause disease^41^. The patient of this report had a daughter and a grandson with cutaneous manifestations (café-au-lait spots on the trunk and limbs, numerous skin neurofibromas, and freckles in the inguinal and axillary regions) highly suggestive of NF1. Unfortunately, it was not possible to carry out the genetic sequencing of both relatives.

The genetic panel also found a mutation in the *POLE* gene. The *POLE* gene is associated with an autosomal-dominant predisposition to colonic adenomatous polyps and colon cancer^4,26^and autosomal recessive FILS syndrome (facial dysmorphism, immunodeficiency, livedo, and short stature) (MedGen UID: 767490). Not all variants present in the *POLE* gene cause disease, but mutations of the *POLE* gene have been associated with a multi-tumor phenotype^33^. The clinical significance of the variant(s) identified in this gene is uncertain. Until this uncertainty is resolved, caution should be exercised before using this result to inform clinical management decisions. In this report, the genetic panel in the patient identified the presence of a mutation in exon 14, c.1370C>T (p.Thr457Met), in heterozygosis that was classified as a VUS. This variant has not been reported in the literature in the germline of individuals with POLE-related conditions. Algorithms developed to predict the effect of missense changes on protein structure and function are either unavailable or do not agree on the potential impact of this missense change. The available evidence is currently insufficient to determine the role of this variant in the disease. Therefore, it has been classified as a VUS. The panel did not identify mutations of the *RET* gene moving away from the diagnosis of MEN2 syndrome.

To the best of our knowledge, this is the first description of an association of an *NF1* pathogenic mutation and VUS in the *POLE* gene in a patient with a previous diagnosis of MTC without mutation of the *RET* gene. However, it is important to highlight that the patient in the present report, in addition to colon PNs and concomitantly a NET, had undergone total thyroidectomy 8 years before an MTC, a multi-tumor phenotype presented in pathogenic mutations of the *POLE* gene. As this variant is not present in population databases and has not been reported in the literature in the germline of individuals with POLE-related conditions, it is difficult to ensure that this variant cannot be related to the multiplicity of tumors presented by the patient in this report.

## CONCLUSIONS

NF1 is a rare genetic disease that can cause various benign or malignant tumors. The association of MTC, PNs, and NET was previously unknown in patients with NF1. The use of a genetic panel in patients with NF1 and other types of neoplasia is a powerful tool to better understand the genotype and phenotype relationships involved in this rare tumor association, and it can identify new mutations.
